# Treatment-Related Factors Affecting the Success of Endodontic Microsurgery and the Influence of GTR on Radiographic Healing—A Cone-Beam Computed Tomography Study

**DOI:** 10.3390/jcm12196382

**Published:** 2023-10-06

**Authors:** Daniel Bieszczad, Jarosław Wichlinski, Tomasz Kaczmarzyk

**Affiliations:** 1 NZOZ Centrum Stomatologii s.c. Justyna Wichlinska, Jaroslaw Wichlinski, ul. 3-go Maja 16, 38-300 Gorlice, Poland; danbiesz@poczta.onet.pl (D.B.); wichlinski@gmail.com (J.W.); 2 Chair of Oral Surgery, Institute of Dentistry, Medical College, Jagiellonian University, ul. Montelupich 4, 31-155 Krakow, Poland

**Keywords:** prognostic factors, endodontic microsurgery (EMS), guided tissue regeneration (GTR), cone-beam computed tomography, PENN 3D criteria

## Abstract

The primary objective of this retrospective study was to assess the correlation between treatment-related factors (resection angle, depth of retrograde filling, length of resected root and use of guided tissue regeneration—GTR) evaluated using cone-beam computed tomography (CBCT) scans and the treatment outcomes of endodontic microsurgery (EMS). The secondary purpose of this research was to evaluate the influence of the GTR technique on the radiographic healing state, taking into account the initial parameters of periapical lesions. In 161 cases, the local factors (volume of a lesion, bone destruction pattern, presence/absence of cortical bone destruction) were measured using preoperative CBCT images before undergoing EMS. At least one year after surgery, the outcome of EMS was classified as a success or a failure (based on radiographic and clinical criteria). Using postoperative CBCT, treatment-related factors (resection angle, depth of retrograde filling, and length of resected root) were measured. Additionally, the status of radiographic healing was evaluated (in accordance with modified PENN 3D criteria). Eighteen cases (11.18%) were classified as failures, and 143 were classified as successes (88.82%). Univariate analysis showed that there was no statistically significant influence of treatment-related factors on the healing outcome of EMS. An exact Fischer’s test showed the significant impact of GTR on radiographic healing (*P* < 0.001) in apical lesions (*P* < 0.001), lesions with a volume between 100 mm^3^ and 450 mm^3^ (*P* < 0.009) and over 450 mm^3^ (*P* < 0.001), lesions with the destruction of one plate (*P* < 0.001), and lesions with the destruction of two plates (through and through) (*P* = 0.022). The use of GTR in apical lesions, lesions with volumes over 100 mm^3^, and lesions with the destruction of at least one plate is significantly associated with better radiographic healing.

## 1. Introduction

In recent decades, endodontic surgery evolved into endodontic microsurgery. This was due to the development of treatment techniques involving magnification, high-power illumination, ultrasonic root-end preparation, microinstruments, and the introduction of biocompatible materials. The advantages of microsurgical techniques are associated with reduced osteotomies (microinstruments, operating microscope, and ultrasonic root-end preparation), easier identification of root apices and meticulous inspection of resected root plane (operating microscope with its illumination and magnification), shallower resection angles (ultrasonic root-end preparation), more accurate root-end fillings (biocompatible materials and operating microscope), and lower postoperative morbidity (microinstruments, operating microscope) [1]. An 80–90% success rate has been reported for primary EMS [2,3,4,5]. Even for secondary EMS, the literature data still show a high success rate [6]. The introduction of cone-beam computed tomography (CBCT) was a key factor impacting the increased success rate of EMS [2]. CBCT is used for the presurgical assessment of the extent of an inflammatory lesion and the degree of destruction of cortical plates. The technique can ascertain the relationship between the lesion and adjacent anatomical structures, the relationship between the roots of adjacent teeth in the area of inflammatory lesion, the cause of the disease itself, and the local parameters that serve as potential prognostic factors in EMS [2,7]. Eventually, this method is used to perform a postoperative assessment of the healing process.

The data collected from CBCT (e.g., dimensions of a lesion, bone destruction pattern, height of the buccal bone plate, and presence/absence of cortical bone destruction) allow the exclusion of cases with poor prognosis, thus increasing the EMS success rate. Although this rate is very high [2,3,4,5], there is still a deficiency in evidence-based data regarding the local parameters affecting the success of surgical treatment [8]. These are divided into three categories: patient-related, tooth-related, and treatment-related factors [8,9]. In our previous report [2], we tested patient- and tooth-related factors using EMS, finding that worse outcomes may be associated with molar teeth, apicomarginal lesions, preoperatively symptomatic lesions and lesions of large apicomarginal dimensions [2]. Scientific data on treatment-related factors in EMS are scarce, and the existing evidence is ambiguous.

One of the most well-studied treatment-related factors is the root-end filling material [9,10]. Some studies show the advantage of using primary EMS over secondary procedures [9,11] and how superior results can be obtained with the use of ultrasonic microtips over burs [10,12]. The effect of GTR on the outcome of EMS is uncertain, as the results of some studies indicate its beneficial influence in “apicomarginal” and “through and through” lesions [2,13,14,15], whereas others report the absence of any effect, especially in lesions with bone defects confined to the periapical region [16,17]. Similarly, regarding EMS of “large lesions>10 mm”, the results of some reports linked better outcomes to EMS involving GTR [2,18]; however, studies with different conclusions are also available [19]. Furthermore, the effect of the resection angle on apical microleakage and the success of EMS has yet to be evaluated in detail. Some studies have also shown that the resection angle has a major impact on the outcome of EMS [1,20,21,22], but other authors have demonstrated that this angle is not as important as the adequate depth of retrograde filling and the application of biocompatible root-end filling material [21,22,23,24]. Conversely, some studies have reported that the depth of the root-end filling does not always result in superior healing success [5,11,25]. There is also no complete agreement as to the distance of resection needed to satisfy biological principles [1,21,26,27]. To the best of our knowledge, there are no CBCT-based studies assessing the influence of GTR on the status of radiographic healing (changes in bone quality and quantity during the regenerative process).

Accordingly, the rationale behind the current study was to evaluate the correlation between CBCT-assessed treatment-related factors (the angle of resection, the depth of retrograde filling, the extent of resection, and the use of GTR) and the treatment outcomes of EMS. The secondary objective was to assess the influence of GTR on the status of radiographic healing.

## 2. Materials and Methods

### 2.1. Data Collection

To evaluate the endpoints of the current study, we partially employed the method used in our previous investigation of patient- and tooth-related factors in EMS [2]. Granted approval no. 1072.6120.192.2021 by the Bioethical Committee of the Jagiellonian University of Krakow, Poland, all data for the current study were retrospectively collected from a subpopulation of patients who received EMS consecutively at a single dental clinic (NZOZ Centrum Stomatologii s.c. Justyna Wichlinska, Jaroslaw Wichlinski, Gorlice, Poland) between March 2015 and December 2021. During this period, 161 teeth in 130 patients were subjected to EMS and were subsequently included in the present study. Patient teeth were categorized into four groups: maxillary anterior, maxillary posterior, mandibular anterior, and mandibular posterior. The group of anterior teeth included incisors and canines, and the posterior group included premolars and molars.

### 2.2. Endodontic Treatment

Out of all 161 teeth assessed, 117 teeth underwent endodontic treatment in our clinic (62— primary endodontic treatment, 55—retreatment), and 44 were treated endodontically elsewhere. All endodontic procedures in our clinic were performed in line with the current standards of endodontic treatment (operating microscope, rubber dam, rotary instruments, ultrasonics, and vertical condensation of gutta-percha). Teeth were not subjected to retreatment in the case of a patient’s disagreement regarding the removal of prosthetic reconstruction, perforations, resorptions, the position of posts being incorrect (with high risk of perforation) or the presence of large posts with thin root canal walls (weakening the structure of the root). Patients were called for follow-up visits 6–12 months after endodontic treatment (both primary and retreatment). In the absence of any signs of radiographic healing, the lesion was rated as persistent and subjected to EMS. Lesions with signs of healing were subjected to further follow-up.

### 2.3. Inclusion/Exclusion Criteria

Inclusion criteria included teeth with persistent periapical lesions after endodontic treatment and preoperative as well as postoperative CBCT (taken at least one year after the surgery).

The exclusion criteria included teeth with fractures or cracks revealed on CBCT images (due to their negative impact on the treatment outcome), increased mobility (II/III), and patients with a history of medication with cytostatic or antiresorptive drugs (due to the risk of osteonecrosis).

### 2.4. Surgical Procedure

Apical surgery was performed with the use of the microsurgical approach by one dentist (D.B.). An operating microscope (Leica M320, Leica Microsystems, Heerbrugg, Switzerland) was used for the inspection of the surface of the resected root, searching for cracks, fractures, isthmuses or additional canals (with the aid of staining using methylene blue), and for retrograde filling, which was performed using the MAP system^ⓡ^ (Produits Dentaires SA, Vevey, Switzerland) after root-end cavity preparation with the use of Piezosurgery^ⓡ^ (Mectron, Carasco, Italy) and ultrasonic microtips. For the root-end filling, MTA+ (Cerkamed, Stalowa Wola, Poland) was used. Piezosurgery^ⓡ^ was also used to enucleate and debride the pathological tissue and smooth the surface of the resected root. The remaining part of the surgery was performed with the use of loupes. In cases of apicomarginal, “through and through” and >10 mm lesions, GTR was used (BioCover^ⓡ^ used as a resorbable collagen membrane to cover the Graft^TM^ as a bone substitute; both by Purgo Biologics, Korea). Owing to the commercial nature of our dental centre and occasional financial limitations of some patients, GTR was used with no protocol.

### 2.5. CBCT Images and Linear Measurement Details

All CBCT images were obtained before and 1 year after EMS. The CBCT images were obtained with the use of CS 8100 3D (Carestream Dental^ⓡ^) at a resolution of 150 microns. Linear measurements were performed on CBCT images by one clinician with 18 years of experience (D.B.) using Carestream dental imaging software. All linear measurements were taken twice (with at least 4-week intervals), and the mean of the two measurements was used for subsequent statistical analyses.

### 2.6. Assessment Criteria

The follow-up visit took place at least one year after the surgical procedure. To assess the treatment outcome of EMS, clinical and radiographic records were analyzed. Clinical evaluation included the assessment of any of the following signs and symptoms: tenderness on palpation or percussion, loss of function, tooth mobility, periodontal pocket, and sinus tract formation. Radiographic healing was qualitatively evaluated by two clinicians (D.B. and J.W.) according to modified PENN 3D criteria [28] using CBCT scans performed 1 year after the surgery. Any discrepancies between the evaluations were settled by discussion. If an agreement could not be reached, advice was sought from a third party (T.K.). Radiographic healing was classified as complete, incomplete, uncertain, or unsatisfactory healing [28]. In turn, the outcome was classified as a success or a failure:

- Success: radiographic healing graded as “complete healing” or “incomplete healing” with no clinical signs or symptoms (tenderness on palpation or percussion, loss of function, tooth mobility, periodontal pocket, sinus tract formation) during the follow-up period (at least 12 months);
- Failure: radiographic healing graded as “uncertain healing” or “unsatisfactory healing” and/or the confirmation of any of the clinical signs or symptoms (tenderness on palpation or percussion, loss of function, tooth mobility, periodontal pocket, sinus tract formation) during the follow-up period (after at least 12 months).

### 2.7. Description of Studied Factors

The following factors were measured using the presurgical CBCT scans:

1. The volume of a lesion (calculated using ITK-SNAP-free software under the GNU General Public Licence) [28,29];
2. Bone destruction pattern (rated in all sections);
3. Presence/absence of cortical bone destruction (rated in all sections).

**Figure 1:**
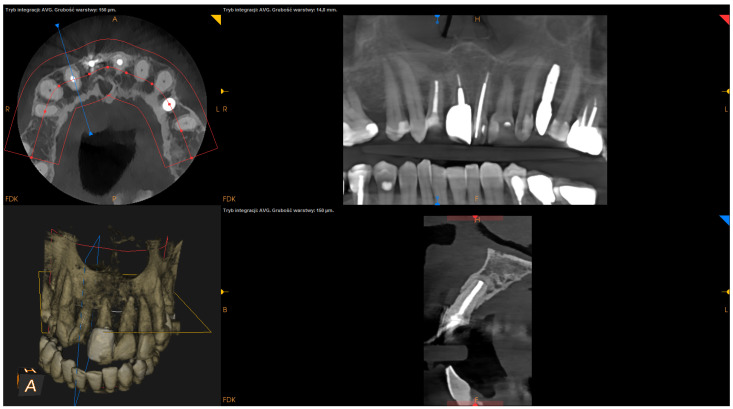
CBCT image (CS 8100 3D Carestream Dental^ⓡ^) example of complete healing based on PENN 3D criteria. Non-English annotations present software technicalities (AVG integration mode and layer thickness).

**Figure 2:**
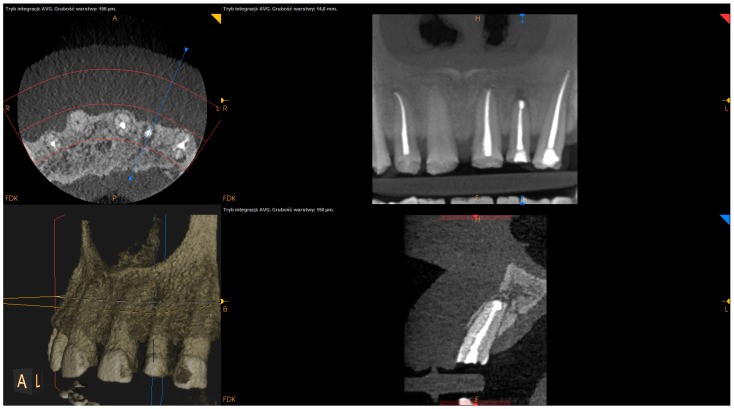
CBCT image (CS 8100 3D Carestream Dental^ⓡ^) example of incomplete healing based on PENN 3D criteria. Non-English annotations present software technicalities (AVG integration mode and layer thickness).

**Figure 3:**
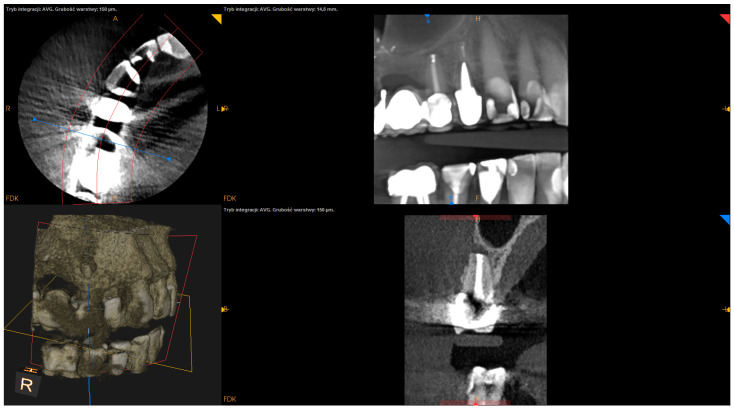
CBCT image (CS 8100 3D Carestream Dental^ⓡ^) example of uncertain healing based on PENN 3D criteria. Non-English annotations present software technicalities (AVG integration mode and layer thickness).

**Figure 4:**
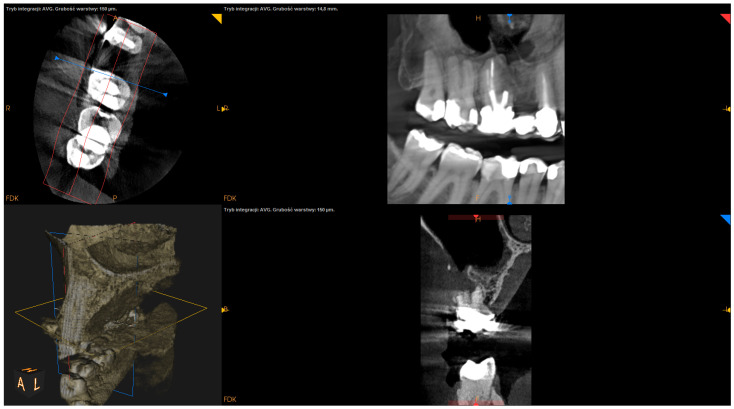
CBCT image (CS 8100 3D Carestream Dental^ⓡ^) example of unsatisfactory healing based on PENN 3D criteria. Non-English annotations present software technicalities (AVG integration mode and layer thickness).

The following factors were measured using the postsurgical CBCT scans:

1. The angle of resection (measured between the resection plane and the long axis of the root; this value was subtracted from 90°);
2. The depth of retrograde filling (measured between the most coronal part of retrofilling and the middle of the most “apical” part of retrofilling in the sagittal section);
3. The extent of resection (measured as the difference between the length before and after resection in the sagittal section);
4. The status of radiographic healing (rated in all sections, according to modified PENN 3D criteria [28]) was classified as complete healing (Figure 1), incomplete healing (Figure 2), uncertain healing (Figure 3), or unsatisfactory healing (Figure 4).

Additionally, patient gender and age, tooth group, and time elapsed from surgery to follow-up were collected. In cases where GTR was used, its impact on radiographic healing and on the treatment outcomes was analyzed.

### 2.8. Statistical Analysis

All statistical analyses were performed using the R Project for Statistical Computing [ver. 4.1.0] [30]. Univariate analyses describing the influence of variables on dichotomous outcomes (success/failure) were performed utilizing the logistic regression model. The results are presented as ORs (odds ratios) with 95% confidence intervals (CIs). Quantitative variables were analyzed by calculating the mean with standard deviation as well as the median with quartiles. Qualitative variables were analyzed by calculating the number of occurrences and percentage rate of occurrence for each value. A comparison of the values of qualitative variables in groups was performed using the chi-square test or Fischer’s exact test, where unexpected frequencies appeared in the tables. The interexaminer agreement of radiographic healing was assessed using the Cohen kappa coefficient. The mean kappa value for qualitative variables was 0.885 (95% CI 0.819–0.951), which demonstrates a high level of agreement between the two examiners (an agreement over 0.8 is considered high). In all tests, the level of significance was set at 0.05.

## 3. Results

**Figure 5:**
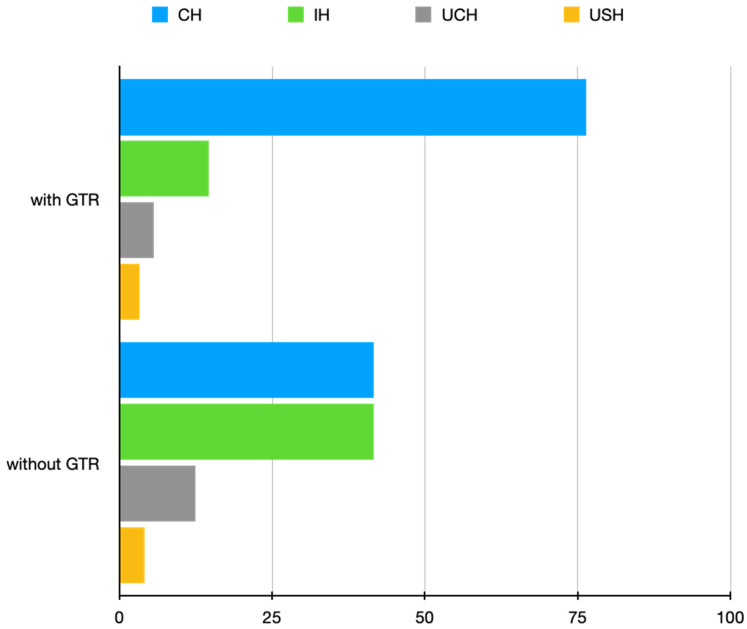
Bar chart of the influence of GTR on radiographic healing. CH: complete healing; IH: incomplete healing; UCH: uncertain healing; USH: unsatisfactory healing.

In all, the complete data of 161 roots were entered into the analysis. The overall success rate was 88.82% (143 out of 161). Table 1 presents the distribution of cases according to demographic parameters, follow-up period, teeth group, treatment-related variables, and outcome. Table 2 shows the distribution of cases according to patient-related and tooth-related parameters. None of the variables reached the level of statistical significance. Table 3 and Table 4 present the univariate logistic regression model of treatment-related factors; none of the parameters reached the level of statistical significance. Table 5 presents the distribution of cases according to the complexity of GTR/preoperative variables of a lesion/radiographic healing state, whereas Table 6 presents the influence of using GTR on the radiographic healing state. The use of GTR had a significant effect on radiographic healing (Figure 5). Subsequent analyses of the influence of GTR on radiographic healing with regard to the bone destruction pattern, the volume of a lesion and the presence/absence of cortical bone destruction are presented in Table 7, Table 8 and Table 9.

GTR had a significant effect on radiographic healing in cases of apical lesions (*P* < 0.001; Fisher’s exact test), lesions with a volume of 100-^450 mm3^ (*P* = 0.009; Fisher’s exact test), lesions with a volume over ^450 mm3^ (*P* < 0.001; Fisher’s exact test), lesions with the destruction of one plate (*P* < 0.001; Fisher’s exact test) and lesions with the destruction of two plates (*P* = 0.022; Fisher’s exact test).

## 4. Discussion

Contrary to our previous report that addressed patient- and tooth-related factors in EMS [2], the aim of the current study was to evaluate the influence of GTR on radiographic healing (which has not been extensively assessed in other studies), taking into account presurgical parameters of periapical lesions (volume, bone destruction pattern, and condition of the cortical bone), as well as to investigate the correlation between treatment-related factors and EMS outcomes.

CBCT analysis allows for a more precise evaluation of a periapical lesion and radiographic healing following EMS than periapical film [28,31,32]. Its main limitation is radiation exposure, but this is outweighed by the ability to meticulously assess the healing process, which is impossible in conventional radiographs [2,33]. In the current study, as well as in our previous report [2], we obtained very high levels of agreement between the examiners in the assessment of qualitative variables, corroborating that CBCT is a very valuable tool for the evaluation of the periapical healing process. EMS requires regular follow-up in order to monitor its evolution. A 1-year follow-up is commonly accepted as an appropriate time span in which to appraise the final outcome [9,34,35]. To the best of our knowledge, no investigation into the EMS outcomes, taking into account the radiographic assessment of the healing process according to modified PENN 3D criteria following surgery with or without GTR, has been published. By employing univariate analysis, we failed to show any significant influence of treatment-related factors on the healing outcome of EMS. However, we demonstrated that GTR had a significant impact on radiographic healing, particularly with regard to apical lesions, lesions with a volume over 100 mm^3^, and lesions with the destruction of at least one cortical plate.

In the current study, we showed the significant differences in the radiographic healing state between procedures performed with or without the GTR technique. Successful healing after EMS depends not only on the bacteria-tight seal of the root canal system with root-end filling but also on the maintenance of the periapical and marginal bone tissue adjacent to the lesion [36]. The aim of GTR is to aid the healing process and bone regeneration, thus providing more successful and predictable outcomes. It directs cell growth towards specific areas of the periodontium damaged by periodontal disease or endodontic pathology [37]. Bone grafts and membranes encourage the growth of key surrounding tissues while protecting the area of regeneration from unwanted cell types, such as epithelial cells [38], which is of particular importance in “apicomarginal lesions” and “through and through” lesions.

In previous studies [17,39,40,41,42], there was no consensus on the use of GTR in four-wall defects. Some studies [17,39,40] showed no beneficial effect regarding the rate of healing of such defects, which is in line with the current results. The application of bone grafts and membranes in these cases has even been criticized as an unnecessary cost [39]. On the other hand, Torres et al. [41] and Dominiak et al. [42] demonstrated that the combined GTR technique allows for a greater success rate in four-wall defects. 

Despite limited evidence, there seems to be a consensus on the use of the GTR technique in the treatment of “large lesions”, “apicomarginal lesions” and “through and through” lesions [2,13,14,15,18,19,33,37,41]. Similar to our previous report [2], in the present study, we showed no significant difference between the treatment outcomes of cases treated with or without the use of GTR. However, one may speculate that, in view of the fact that GTR was used in complicated cases, the difference might be significant if these cases were treated without GTR. However, this requires confirmation in large-scale studies. Regardless of the equivocal effects of GTR demonstrated in relation to “large lesions”, “apicomarginal lesions” and “through and through” lesions in previous reports [13,43], the current study showed significantly superior radiographic healing in these instances. In “large lesions”, “apicomarginal lesions” and “through and through” lesions, where the periosteum is likely to be damaged by the inflammatory process, extensive periapical bone destruction tends to be replaced by fibrous connective tissue (scar tissue formation), giving the effect of “incomplete healing” [2,33,43]. Despite the fact that the majority of lesions in the current series had a volume >100 mm^3^ (with a mean of 581.72 mm^3^) and exhibited the destruction of one or two plates, in cases where GTR was used, we observed “complete healing” considerably more often than “incomplete healing” in the assessment of radiographic healing status according to the PENN 3D criteria (Figure 5). This clearly indicates an increase in bone quantity and quality after using GTR in the treatment of complicated cases. Additionally, upon the evaluation of CBCT images, we also noticed that the use of GTR prevented bone collapse, which represents another advantage of using GTR. These results show the validity of the use of GTR in complicated cases as they help surgeons with treatment decisions and finally allow for a more unequivocal assessment of treatment outcomes. The results of our study, employing the PENN 3D criteria in the detailed assessment of radiographic healing in the context of GTR use, may start a new chapter in assessing the impact of GTR on the radiographic healing process.

The effect of the resection angle on healing outcomes has not been evaluated in detail in most of the published material. Although traditional apical surgery recommends a resection angle of 30–45°, currently, there is a consensus that this angle should be as perpendicular to the long axis of the root as possible [1,20]. This is because the acute resection angle does not allow the removal of all apical ramifications, increases the number of patent dentinal tubules, elongates the outline of the root canal and, according to some authors [21,22], increases the amount of leakage of bacteria and toxins. Some authors reported no association between the angle and leakage [23,24]; however, they applied MTA as a root-end filling material whose properties might have counteracted the limitations of acute beveling. Additionally, the in vitro study by Garip et al. [23] showed that, in the case of adequate retrograde cavity depth preparation, the variation in the angle value does not necessarily cause any difference in leakage and therefore may not have any significant effect on the treatment outcome, which is in line with the current results. 

In the paper by von Arx T. et al. [20], roots with a shallow resection angle (<20°) had a higher (though insignificant) rate of success than those with an acute resection angle (>20°). In turn, Villa-Machado et al. [44] showed a significant difference between failures in teeth with minimal and pronounced bevel in univariate analysis. However, in multivariate analysis, the difference proved to be insignificant [44]. In the current study, we failed to demonstrate any impact of beveling on the treatment outcome, without even noticeable differences between the subgroups in terms of treatment outcomes, unlike in previous studies [20,44]. However, the mean angle in the current series was 6.02°, as opposed to 17.7° in the study by von Arx T. et al. [20]. Accordingly, the angle in the current series was closer to the model angle of 0°. One may speculate that some slight deviations from the 0° angle do not result in noticeable differences in the healing outcome, particularly when an adequate depth of retrograde filling (at least reaching the level of the most coronal aspect of the beveled root-end plane) was achieved with a biocompatible material. These factors are of the utmost importance in decreasing apical microleakage [21,22,23,24] where, due to difficult local conditions (sharply angled teeth, abundant mental protuberance), it is impossible to achieve a 0° resection angle.

One of the key points for success in EMS is the need for the root canal system to be tightly sealed following root-end resection in order to minimize the risk of apical leakage. Microleakage is a sum of two pathways: one along the interface between the retrofilling material and the canal wall, and a second along open dentinal tubules at the resected root-end. The results of most of the studies indicate that the depth of retrograde filling required to produce a safe and effective seal is between 3.0 mm and 3.5 mm [21,22]. In turn, the results of a Toronto study [11] showed that nonsurgical retreatment followed by EMS without retrograde filling is an acceptable alternate treatment, especially in roots that have undergone modern endodontic treatment with extensive resection. This approach, however, may be an option in treating anterior teeth with less complex anatomies than found in posterior teeth.

In the current series, we failed to demonstrate any significant impact of the depth of retrograde filling on the outcome of EMS. Similarly, von Arx T. et al. [45] did not show any statistical effect of the depth of retrograde filling on the healing outcome. The mean depth of retrograde filling in their study was similar to ours (2.02 mm and 2.11 mm, respectively). In view of the percentage of success achieved in the present study, with a noticeable (however insignificant) improvement in prognosis accompanying an increase in the depth of retrofilling, one may speculate that a depth of 2 mm is enough to form an effective seal (considering the overall results of EMS) in roots after modern endodontic treatment using biocompatible retrofilling material (MTA).

With regard to cases with no retrograde filling (78.57% of success in our study), there may be some benefits to this solution. However, this is only in cases subjected to modern endodontic treatment, especially in anterior teeth with very difficult local conditions (sharply angled teeth, abundant mental protuberance) where ultrasonic preparation of retrograde cavity could cause more complications (microcracks, axis of retrograde cavity not longitudinal to the axis of the canal) than benefits.

Another variable studied in the current series was the extent of resection. Some authors suggest resecting at least 2 mm of the apex [21], but most studies indicate the necessity of a 3 mm resection, which is due to the presence of most ramifications and lateral canals in that range of the apex [1,26,27]. In the present study, we did not find any significance in this regard. It is possible that some degree of resection over 3 mm may somewhat aggravate the prognosis. However, we believe that such a distance is often related to apicomarginal lesions (with possible microcracks or fractures), with worse initial prognosis [2]. Thus, the prognosis is not always correlated with the extent of resection (e.g., in long roots of canines, it may even be more than 5 mm without impairing the prognosis). From personal experience, however, in cases of resorptions, posts or short roots, the length of resection may be shorter. For these reasons, in certain circumstances, the clinical judgement and experience of a surgeon may be more valuable than strict adherence to the 3 mm rule. We believe that the experience of a surgeon may play a pivotal role in making decisions about variables such as resection angle, depth of retrograde filling or resection length, especially in more complex cases. 

In the current study, there was a noticeable (although insignificant) decrease in the survival rates of cases with a follow-up over 4 years. This was unlike the results in the study by Grung et al. [46], where long-term follow-up showed an overall success rate of 87.2% vs. 80.9% at the 1-year follow-up appointment. In our opinion, the time-dependent decline in survival rates may not necessarily be associated with the failure of EMS but can be a natural consequence of other complications typical of endodontically treated teeth (especially root fractures or the failure of prosthetic reconstruction).

The success rate of all surgeries in this series was almost 89%, which is comparable with the results of other studies [2,3,4,5]. The strength of this single-operator study is the minimalization of intraoperative variations related to the surgical procedure [47]. However, this report is not free from shortcomings related to some technical habits and unintentional mistakes related to a single operator, which might be repeated [2], as well as to the small study sample. In addition, because of the retrospective nature of this study, it was impossible to fully standardize the treatment protocols. We were unable to employ the strict protocol of GTR use. Hence, the current results require confirmation in prospective studies with specific criteria. Specifically, we should compare the state of radiographic healing with the results of histological evaluation.

## 5. Conclusions

In summary, the use of GTR in apical lesions, lesions with volumes over 100 mm3, and lesions with the destruction of at least one cortical plate is associated with significantly better radiographic healing. The resection angle, depth of retrograde filling, extent of resection and GTR have no marked impact on healing outcomes. CBCT should be considered a mandatory step in the postsurgical evaluation of the healing progress of EMS.

## Figures and Tables

**Table 1:** Descriptive statistics of the study sample and treatment-related factors.

Parameter		*n* = 161
**Age [years]**	mean ± SD	43.3 ± 10.87
	median (range)	43 (36–51)
**Gender**	F	76 (47.20%)
	M	85 (52.80%)
**Follow-up period [years]**	mean ± SD	3.17 ± 1.95
	median (range)	2 (2–5)
**Teeth group**	maxillary anterior	89 (55.28%)
	maxillary posterior	48 (29.81%)
	mandibular anterior	15 (9.32%)
	mandibular posterior	9 (5.59%)
**Extent of resected root [mm]**	mean ± SD	2.83 ± 1.36
	median (range)	2.5 (2–3.5)
**Depth of retrograde filling [mm]**	mean ± SD	2.11 ± 0.87
	median (range)	2.5 (1.5–2.5)
**Resection angle [°]**	mean ± SD	6.02 ± 10.72
	median (range)	4 (−1–12) *
**GTR**	with GTR	89 (55.28%)
	without GTR	72 (44.72%)
**Outcome**	success	143 (88.82%)
	failure	18 (11.18%)

* negative values = resection plane inclined towards the lingual/palatal aspect; positive values = resection plane inclined towards the labial/buccal aspect [20]. SD: standard deviation; F: female; M: male; GTR: guided tissue regeneration; n: sample size.

**Table 2:** Distribution of cases per preoperative variable and outcome; univariate logistic regression model.

Parameter		Success	OR (95% CI)	*P* Value Univariate Logistic Regression
**Gender**	F (*n* = 76)	68 (89.47%)	1.00	
	M (*n* = 85)	75 (88.24%)	0.882 (0.329–2.365)	0.803
**Age [years]**	<40 years (*n* = 64)	59 (92.19%)	1.00	
	40 < 60 years (*n* = 85)	75 (88.24%)	0.636 (0.206–1.96)	0.43
	>60 years (*n* = 12)	9 (75.00%)	0.254 (0.052–1.252)	0.092
**Teeth group**	mandibular anterior (*n* = 15)	14 (93.33%)	1.00	
	maxillary anterior (*n* = 89)	83 (93.26%)	0.988 (0.11–8.841)	0.991
	mandibular posterior (*n* = 9)	8 (88.89%)	0.571 (0.031-10.434)	0.706
	maxillary posterior (*n* = 38)	38 (79.17%)	0.271 (0.032–2.319)	0.233
**Follow-up period [years]**	<2 years (*n* = 85)	77 (90.59%)	1.00	
	3–4 years (*n* = 28)	25 (89.29%)	0.866 (0.213–3.516)	0.84
	>4 years (*n* = 48)	41 (85.42%)	0.609 (0.206–1.797)	0.369

OR: odds ratio; CI: confidence interval; F: female; M: male; n: sample size.

**Table 3:** Univariate logistic regression model of treatment-related variables (with no division into subgroups).

Parameter	OR (95% CI)	*P* Value Univariate Logistic Regression
**Resection angle**	0.998 (0.953–1.044)	0.915
**Depth of retrograde filling**	1.511 (0.903–2.529)	0.116
**Extent of resected root**	0.755 (0.551–1.036)	0.082

OR: odds ratio; CI: confidence interval.

**Table 4:** Distribution of cases per treatment-related variables (with the division into subgroups) and outcome; univariate logistic regression model.

Parameter		Success	OR (95% CI)	*P* Value Univariate Logistic Regression
**Resection angle**	<1° (*n* = 59)	52 (88.14%)	1.00	
	2° < 10° (*n* = 54)	75 (90.74%)	1.319 (0.393–4.433	0.654
	>10° (*n* = 48)	9 (87.50%)	0.942 (0.294–3.017)	0.92
**Depth of retrograde filling**	0 mm (*n* = 14)	11 (78.57%)	1.00	
	1–1.5 mm (*n* = 29)	25 (86.21%)	1.705 (0.325–8.933)	0.528
	2–2.5 mm (*n* = 85)	76 (89.41%)	2.303 (0.539–9.834)	0.26
	>2.5 mm (*n* = 33)	31 (93.94%)	4.227 (0.622–28.743)	0.14
**Extent of resected root**	<2 mm (*n* = 61)	56 (91.80%)	1.00	
	2.1 < 3 mm (*n* = 57)	53 (92.98%)	1.183 (0.301–4.643)	0.81
	>3 mm (*n* = 43)	34 (79.07%)	0.337 (0.104–1.09)	0.069
**GTR**	with GTR (*n* = 89)	81 (91.01%)	1.00	
	without GTR (*n* = 72)	62 (86.11%)	0.612 (0.0228–1.643)	0.33

OR: odds ratio; CI: confidence interval; GTR: guided tissue regeneration; n: sample size.

**Table 5:** Descriptive statistics of the complex: GTR/preoperative variables of a lesion/radiographic healing state.

Parameter		*n* = 161
**GTR**	with GTR	89 (55.28%)
	without GTR	72 (44.72%)
**Bone destruction pattern**	apical	126 (78.26%)
	apicomarginal	35 (21.74%)
**Volume of a lesion [mm^3^]**	mean ± SD	581.72 (847.41)
	median	267 (104–632)
	range	15–5127
**Presence/absence of cortical bone destruction**	no destruction	62 (38.51%)
	destruction of one plate	84 (52.17%)
	destruction of both plates (through and through lesions)	15 (9.32%)
**Radiographic healing state**	CH	98 (60.87%)
	IH	43 (26.71%)
	UCH	14 (8.70%)
	USH	6 (3.73%)

CH: complete healing; IH: incomplete healing; UCH: uncertain healing; USH: unsatisfactory healing; SD: standard deviation; GTR: guided tissue regeneration; n: sample size.

**Table 6:** Descriptive statistics of the influence of GTR on radiographic healing.

Radiographic Healing State	Use of GTR		*P* Value (Fisher’s Exact Test)
	with GTR (*n* = 89)	without GTR (*n* = 72)	
CH	68 (76.40%)	30 (41.67%)	<0.001 *
IH	13 (14.61%)	30 (41.67%)	
UCH	5 (5.62%)	9 (12.50%)	
USH	3 (3.37%)	3 (4.17%)	

Asterisk denotes significance. CH: complete healing; IH: incomplete healing; UCH: uncertain healing; USH: unsatisfactory healing; n: sample size.

**Table 7:** Descriptive statistics of the influence of GTR on radiographic healing in relation to the bone destruction pattern.

**APICAL (*n* = 126)**			
**Radiographic healing state**	**Use of GTR**		***P* exact Fisher’s test**
	**with GTR (*n* = 69)**	**without GTR (*n* = 57)**	
CH	59 (85.51%)	25 (43.86%)	<0.001 *
IH	8 (11.59%)	26 (45.61%)	
UCH	2 (2.90%)	5 (8.77%)	
USH	0 (0.00%)	1 (1.75%)	
**APICOMARGINAL (*n* = 35)**			
**Radiographic healing state**	**Use of GTR**		***P* exact Fisher’s test**
	**with GTR (*n* = 20)**	**without GTR (*n* = 15)**	
CH	9 (45.00%)	5 (33.33%)	0.9
IH	5 (25.00%)	4 (26.67%)	
UCH	3 (15.00%)	4 (26.67%)	
USH	3 (15.00%)	2 (13.33%)	

Asterisk denotes significance. CH: complete healing; IH: incomplete healing; UCH: uncertain healing; USH: unsatisfactory healing; GTR: guided tissue regeneration; n: sample size.

**Table 8:** Descriptive statistics of the influence of GTR on radiographic healing in relation to the volume of a lesion.

**Volume of a lesion < 100 mm^3^ (*n* = 39)**			
**Radiographic healing state**	**Use of GTR**		***P* exact Fisher’s test**
	**with GTR (*n* = 20)**	**without GTR (*n* = 19)**	
CH	12 (60.00%)	8 (42.11%)	0.385
IH	4 (20.00%)	8 (42.11%)	
UCH	3 (15.00%)	1 (5.26%)	
USH	1 (5.00%)	2 (10.53%)	
**Volume of a lesion between 100 mm^3^ and 450 mm^3^ (*n* = 69)**			
**Radiographic healing state**	**use of GTR**		***P* exact Fisher’s test**
	**with GTR (*n* = 31)**	**without GTR (*n* = 38)**	
CH	24 (77.42%)	5 (47.37%)	0.009 *
IH	5 (16.13%)	4 (34.21%)	
UCH	0 (0.00%)	4 (15.79%)	
USH	2 (6.45%)	2 (2.63%)	
**Volume of a lesion > 450 mm^3^ (*n* = 53)**			
**Radiographic healing state**	**use of GTR**		***P* exact Fisher’s test**
	**with GTR (*n* = 38)**	**without GTR (*n* = 15)**	
CH	32 (84.21%)	4 (26.67%)	<0.001 *
IH	4 (10.53%)	9 (60.00%)	
UCH	2 (5.26%)	2 (13.33%)	
USH	0 (0.00%)	0 (0.00%)	

Asterisk denotes significance. CH: complete healing; IH: incomplete healing; UCH: uncertain healing; USH: unsatisfactory healing; n: sample size; GTR: guided tissue regeneration.

**Table 9:** Descriptive statistics of the influence of GTR on radiographic healing in relation to the state of bone cortices.

**No destruction (*n* = 62)**			
**Radiographic healing state**	**Use of GTR**		***P* exact Fisher’s test**
	**with GTR (*n* = 31)**	**without GTR (*n* = 31)**	
CH	21 (67.74%)	16 (51.61%)	0.382
IH	6 (19.35%)	9 (29.03%)	
UCH	4 (12.90%)	4 (12.90%)	
USH	0 (0.00%)	2 (6.45%)	
**Destruction of one plate (*n* = 84)**			
**Radiographic healing state**	**Use of GTR**		***P* exact Fisher’s test**
	**with GTR (*n* = 46)**	**without GTR (*n* = 38)**	
CH	37 (80.43%)	14 (36.84%)	<0.001 *
IH	6 (13.04%)	19 (50.00%)	
UCH	0 (0.00%)	4 (10.53%)	
USH	3 (6.52%)	1 (2.63%)	
**Destruction of both plates (through and through lesions) (*n* = 15)**			
**Radiographic healing state**	**Use of GTR**		***P* exact Fisher’s test**
	**with GTR (*n* = 12)**	**without GTR (*n* = 3)**	
CH	10 (83.33%)	0 (0.00%)	0.022 *
IH	1 (8.33%)	2 (66.67%)	
UCH	1 (8.33%)	1 (33.33%)	
USH	0 (0.00%)	0 (0.00%)	

Asterisk denotes significance. CH: complete healing; IH: incomplete healing; UCH: uncertain healing; USH: unsatisfactory healing; GTR: guided tissue regeneration; n: sample size.

## Data Availability

The data that support the findings are available on request from the first author, D.B.
